# Is CepA from *Klebsiella pneumoniae* a biocide pump? Evidence suggests a metal efflux function

**DOI:** 10.1128/msphere.00512-25

**Published:** 2025-11-17

**Authors:** Le Phung Hien, Melissa H. Brown

**Affiliations:** 1 College of Science and Engineering, Flinders University, Bedford Park, South Australia, Australia; 2 ARC Training Centre for Biofilm Research and Innovation, Flinders University, Bedford Park, South Australia, Australia; Universita degli Studi di Napoli Federico II, Naples, Italy

**Keywords:** CepA, *Klebsiella pneumoniae*, efflux pump, biocide, heavy metal, antimicrobial resistance

## Abstract

Amid the antibiotic resistance crisis, biocides, including antiseptics and disinfectants, are indispensable tools to limit the spread of nosocomial infections and extensively drug-resistant bacteria. Thus, it is crucial that they remain effective. Extruding biocides from the cell using efflux systems is one mechanism by which bacteria can survive in their presence. The CepA protein from *Klebsiella pneumoniae* belongs to a family of heavy metal exporters but has been reported to be associated with chlorhexidine resistance, a common biocide. This study aims to elucidate the function of CepA as a biocide transporter and clarify its substrate profile in *Escherichia coli*. The results support the role of CepA in the transport of Fe^2+^, Mn^2+^, and possibly Cu^2+^ and Zn^2+^ to a lesser extent. The transporter also contributes to cell tolerance to iron and manganese stress. However, in contrast to previous reports, no significant impact of the expression of this protein on bacterial resistance to quaternary ammonium biocides was detected. Therefore, we propose that the main role of *K. pneumoniae* CepA is as a heavy metal transporter.

**IMPORTANCE:**

Understanding the structure and function of efflux pumps is crucial for addressing efflux-mediated resistance. Transporters that pump out biocides or heavy metals are not uncommon. However, to date, no transport protein capable of extruding both types of substrates has been reported. Initially, it was proposed that *Klebsiella pneumoniae* CepA was associated with chlorhexidine resistance and subsequently considered a biocide efflux pump. That notion also hinted at a novel group of drug efflux pumps that can extrude both biocides and heavy metals, a direct mechanism of cross-resistance between the two. The findings of this study indicate that it is more plausible that *K. pneumoniae* CepA is involved in metal homeostasis rather than in chlorhexidine biocide resistance in the recombinant *Escherichia coli*.

## INTRODUCTION

Due to the dissemination of extensively drug-resistant bacteria and the dwindling antibiotic discovery pipeline, it is widely acknowledged that antibiotic resistance is a global health crisis (1, 2). In 2021, bacterial antimicrobial resistance was the cause of approximately 4.71 million deaths, direct and associated. Predictions based on current trends showed that resistant bacteria will directly cause up to 2.3 million deaths and be associated with up to 9.7 million deaths in 2050, which will overtake cancer and any other unnatural cause of death (3). This crisis has forced the world to rely on alternatives to control infectious diseases, among which the only currently viable and effective options are biosecurity practices and biocides (4, 5).

Biocides have been widely used as antiseptics and disinfectants in healthcare settings (6), in the food and beverage industries (7, 8), as preservatives in various other consumables, and in virtually all personal care and cleaning products (9). Common biocides include alcohol, chlorhexidine, quaternary ammonium compounds, and bleach. Over-the-counter and household products that contain biocides have seen a rapid increase in usage during the last decade, especially during and after the COVID-19 pandemic, despite not being put under strict regulation (10, 11). This trend gives rise to a concern about the lack of biocide stewardship and ensuing resistance (12).

There are many mechanisms by which bacteria can survive biocide exposure. Among them, one of the most versatile and effective mechanisms is to utilize efflux pumps (13). Efflux pumps are membrane transport proteins that can extrude compounds out of the cell. Some of them can efflux a wide structurally diverse range of substrates, including antibiotics and biocides (14). Therefore, understanding the structure and functionality of efflux pumps can encourage the discovery of strategies to inhibit these transporters and potentiate the activity of existing antimicrobials. There are currently seven recognized families/superfamilies of bacterial drug efflux pumps: the ATP-binding cassette family, the major facilitator superfamily, the multidrug and toxin extrusion family, the small multidrug resistance family, the proteobacterial antimicrobial compound efflux family, the p-aminobenzoyl-glutamate transporter family, and the resistance-nodulation-cell division superfamily (15). There are also putative groups of drug efflux pumps whose activity has not been widely verified (16, 17), one of which, the Cation Diffusion Facilitator (CDF) family, is the focus of this study.

*Klebsiella pneumoniae* is a major opportunistic pathogen that causes nosocomial infections (18). Fang et al. (16) reported a novel cation efflux pump gene from a chlorhexidine-resistant *K. pneumoniae* that was proposed to act as a cation efflux pump associated with chlorhexidine resistance. Cloning of the gene into *Escherichia coli* elevated the MIC of tested strains by two- to fourfold. Interestingly, analysis of the gene sequence indicated that it encoded a novel efflux pump protein that belonged to the CDF family, a group of transporters known for heavy metal extrusion activity, with no drug-exported activity reported (19). This report hinted at a novel function of this pump, being able to extrude both biocides and heavy metals, a direct mechanism of cross-resistance between the two. Since the study, there have been reports on the presence of this gene in different gram-negative and gram-positive bacteria, including *K. pneumoniae*, *E. coli*, *Acinetobacter baumannii,* and *Staphylococcus aureus* (20–23); however, no further study has been made on characterizing CepA itself. This study aims to elucidate the function of this putative chlorhexidine efflux pump, CepA from *Klebsiella*, as a biocide transporter, and to clarify its substrate profile in the heterologous host *E. coli*.

## RESULTS

### *Klebsiella* CepA is a Cation Diffusion Facilitator protein closely related to *E. coli* FieF

**Fig 1:**
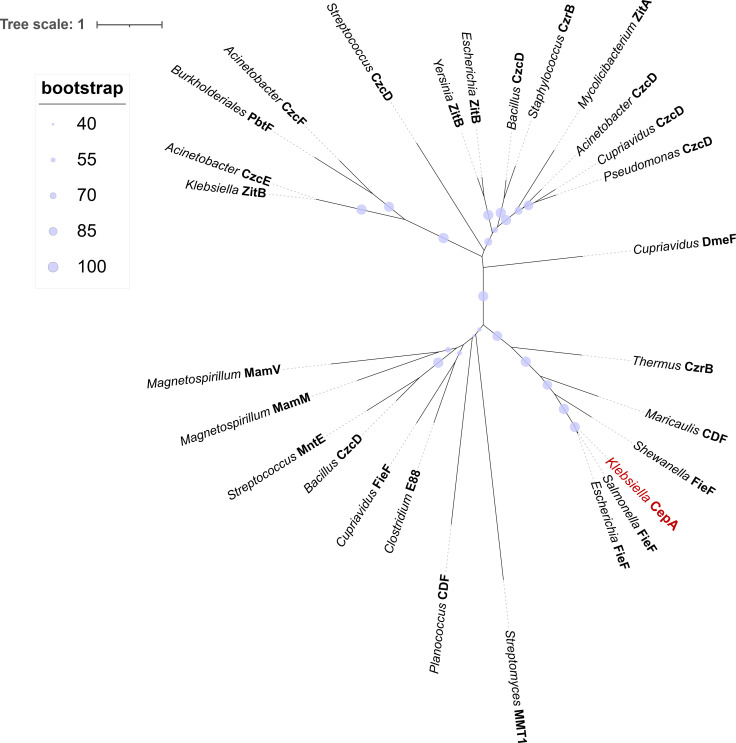
Maximum likelihood phylogenetic tree of *Klebsiella pneumoniae* CepA and selected proteins from the CDF family of proteins. The tree was constructed using IQ-TREE 2.2.0 with automatic model selection for nucleotide substitution, default heuristic search options, and ultrafast bootstrapping with 10,000 replicates. Accession numbers of proteins are listed in Table S1. The bootstrap value is represented at each branch.

To assess the conservation of *cepA* in the core genome of *K. pneumoniae*, a NCBI BLAST search using the *cepA* DNA sequence was conducted, revealing the ubiquitous presence of this gene within *Klebsiella* genomes. To confirm the assignment of CepA into the CDF family of transport proteins, the CepA amino acid sequence was aligned with 27 CDF proteins using Clustal Omega. These representatives were selected as they are diverse in amino acid sequence and originate from different bacterial species (Table S1; Fig. S1). CepA was found to share between 20.6% and 49.3% amino acid sequence similarity with most proteins (Table S1), with the highest being 89% and 88.2% to the FieF protein from *Salmonella* and *E. coli*, respectively. A maximum likelihood phylogenetic tree was constructed using the IQ-TREE program (Fig. 1), which confirmed the initial allocation of CepA to the CDF family (24). CepA was found to form a very tight cluster with FieF proteins from *Salmonella* and *E. coli* with a bootstrap value of 94%, suggesting the close homology between these proteins. Multiple sequence alignments also revealed that the residues involved in metal binding and transportation in *E. coli* FieF (25) are conserved in CepA. The AlphaFold-predicted structure of CepA is highly similar to the crystal structure of FieF with an RMSD of 0.513 (Fig. S2). These data suggest that CepA may be similar to *E. coli* FieF in terms of structure and function.

### CepA was expressed as a membrane protein in different *E. coli* strains

A *cepA* sequence codon-optimized for expression in *E. coli*, including a C-terminal His-tag extension (Fig. S3 and S4), was synthesized and cloned into the vector pBlueScript II SK (+) (pBS) to create pBS-CepA, placing *cepA* under the regulation of the T7 promoter. To compare CepA expression and activity among different *E. coli* strains, pBS-CepA and the pBS empty vector were transformed into *E. coli* strains DH5α, DH5α Δ*acrAB*, BL21(DE3) Δ*acrAB*, and BW25113 Δ*acrAB*. The latter three strains have the major multidrug efflux pump system AcrAB inactivated, rendering the cells hypersensitive to some antimicrobials (26). Additionally, the pBS empty vector was transformed into the parental *E. coli* BL21(DE3) and BW25113 strains, each possessing an intact AcrAB system, to assess the effect of AcrAB deletion on substrate sensitivity. This generated the following *E. coli* strains: DH5α (pBS), DH5α (pBS-CepA), DH5α Δ*acrAB* (pBS), DH5α Δ*acrAB* (pBS-CepA), BW25113 (pBS), BW25113 Δ*acrAB* (pBS), BW25113 ΔAcrAB (pBS-CepA), BL21(DE3) (pBS), BL21(DE3) Δ*acrAB* (pBS), and BL21(DE3) Δ*acrAB* (pBS-CepA), which are subsequently abbreviated to DH-p, DH-C, DHΔ-p, DHΔ-C, BW-p, BWΔ-p, BWΔ-C, BL-p, BLΔ-p, and BLΔ-C, respectively.

To evaluate the expression of *cepA* and ensure that expression levels did not influence functionality, total membrane fractions were isolated from growing cultures, followed by SDS-PAGE and Western blot detection using a His-specific antiserum. A band corresponding to the predicted molecular weight of His-tagged CepA (32.9 kDa) could be detected in all strains carrying pBS-CepA, which was absent in strains only carrying pBS. CepA expression levels differed between *E. coli* carrier strains, with IPTG-induced BLΔ-C exhibiting the highest expression level, followed by its uninduced counterpart (Fig. S5). This level of expression is expected since the BL21(DE3) strain carries the T7 polymerase gene, which is crucial for efficient transcription of T7 promoter-regulated genes. The T7 polymerase gene is regulated by the *lac* promoter, and its leaky nature permits a high basal-level production of T7 polymerase even when uninduced (27). In contrast, DH-C, DHΔ-C, and BWΔ-C strains, which lack the T7 polymerase gene, still exhibited lower expression levels of CepA, possibly due to the presence of a promoter region upstream of the multiple cloning site. This feature is specific to the pBS vector and has been exploited to allow low-level expression of the staphylococcal efflux pump QacA in *E. coli* (28).

Subsequently, strains from the two backgrounds, *E. coli* BWΔ and BLΔ, were selected for further experiments since the expression of CepA in these strains was deemed sufficient for functional analyses. For BLΔ-C, experiments were conducted without IPTG induction to reduce the metabolic burden on the cells and avoid potential IPTG interference in functional assays.

### CepA did not affect the resistance of *E. coli* strains against various metals, biocides, and dyes

To ascertain if CepA plays a role in antimicrobial resistance, BWΔ-p, BWΔ-C, BLΔ-p, and BLΔ-C bacterial cultures were subjected to MIC assays against a range of agents. Besides chlorhexidine, which was reported in the original article (16), common cationic biocides, benzalkonium and pentamidine, together with additional cationic dyes and biocides with diverse structures, were tested. Ethidium was also included since it is a common substrate for the majority of multidrug efflux pumps (29). Comparison between the resistance afforded by BWΔ-p, BW-p, BLΔ-p, and BL-p cultures (Table 1) confirmed that the absence of the AcrAB pump sensitizes the strains to all tested compounds. In general, strains from a BW background exhibit higher MICs than their counterparts from BL backgrounds, possibly due to the difference in genetic backgrounds that is reflected in the basal resistance levels. Irrespective of this, no significant difference in MICs was observed between CepA-expressing strains and strains with the empty vector. These data indicate that CepA, at the levels of expression achieved here in sensitized *E. coli* strains, does not significantly contribute to the biocide resistance of sensitized *E. coli* strains.

**TABLE 1:** The MIC of different biocides and dyes against six bacterial strains

Biocide and dyes	Value	MIC value (µg/mL)*^a^*					
		BW-p	BWΔ-p	BWΔ-C	BL-p	BLΔ-p	BLΔ-C
Benzalkonium	Mode	32	1	1	16	1	1
	Max	32	2	2	16	1	1
Chlorhexidine	Mode	2	0.5	0.5	1	0.5	0.5
	Max	2	1	1	2	0.5	0.5
Ethidium	Mode	128	4	4	64	1	1
	Max	256	4	4	64	1	1
Pentamidine	Mode	256	64	64	64	32	32
	Max	256	64	64	128	32	32
Acridine orange	Mode	256	16	16	64	2	2
	Max	256	16	16	128	2	2
Acriflavine	Mode	64	8	8	16	1	1
	Max	128	8	8	16	1	1
Proflavine	Mode	64	8	8	16	2	2
	Max	64	8	8	16	2	2
Pyronin Y	Mode	16	2	2	8	0.5	0.5
	Max	16	2	2	8	0.5	1
Methyl green	Mode	256	64	64	512	32	32
	Max	512	64	64	512	32	32
Crystal violet	Mode	4	1	1	4	1	1
	Max	4	1	1	4	1	1
Methylene blue	Mode	512	8	8	512	4	4
	Max	512	8	8	512	4	4
Methyl viologen	Mode	256	256	128	128	64	64
	Max	512	256	256	128	64	64

^*a*^ BWΔ-C and BLΔ-C express CepA, BWΔ-p and BLΔ-p contain the empty vector, and BW-p and BL-p have an intact AcrAB system. Tests were conducted in four replicates, with the mode value (the value that appears the most) and maximum value shown.

The CDF family of transporters is known to transport heavy metal ions, notably Fe^2+^, Mn^2+^, Cu^2+^, Zn^2+^, Co^2+^, and Cd^2+^ (30); the first four metals are important trace elements for bacteria, while cadmium and copper are major heavy metals that can pollute the environment. To determine if CepA is involved in metal resistance, thereby sharing a functional similarity with its CDF homologs, BWΔ-p, BWΔ-C, BLΔ-p, and BLΔ-C cells were subjected to MIC assays using these six metal ions.

To achieve this, two liquid media were used to determine the metal resistance capacity of cells with CepA: Luria-Bertani (LB) and HEPES minimal medium (HMM). Rich media have been reported to increase the MIC value of bacteria to metals since high concentrations of sugars, biomolecules, and phosphate can form complexes with metal ions and decrease their toxicity; this effect is least pronounced in LB medium (31). Thus, the HMM medium was also included for comparison, where HEPES was used instead of a phosphate buffer and glycerol as the carbon source instead of glucose to minimize metal binding capacity. The MIC values of individual metal ions against these four strains are presented in Table 2.

**TABLE 2:** The MIC of different metal ions against six bacterial strains^*a*^

Metal ion	Medium		MIC (mM)^*b*^					
			BW-p	BWΔ-p	BWΔ-C	BL-p	BLΔ-p	BLΔ-C
Fe^2+^	HMM	Mode value	n.d.	n.d.	n.d.	n.d.	n.d.	n.d.
		Max value	n.d.	n.d.	n.d.	n.d.	n.d.	n.d.
	LB	Mode value	6	6	6	8	8	8
		Max value	6	6	8	8	8	8
Mn^2+^	HMM	Mode value	n.d.	n.d.	n.d.	n.d.	n.d.	n.d.
		Max value	n.d.	n.d.	n.d.	n.d.	n.d.	n.d.
	LB	Mode value	12	12	12	16	12	12
		Max value	16	12	12	16	12	12
Zn^2+^	HMM	Mode value	0.25	0.375	0.375	0.5	0.75	0.75
		Max value	0.25	0.375	0.375	0.75	0.75	0.75
	LB	Mode value	1.5	2	2	2	2	2
		Max value	2	3	3	2	2	2
Cu^2+^	HMM	Mode value	0.75	0.75	0.75	0.5	0.5	0.5
		Max value	0.75	0.75	0.75	0.5	0.5	0.5
	LB	Mode value	6	6	6	6	6	6
		Max value	6	6	6	6	6	6
Cd^2+^	HMM	Mode value	0.25	0.25	0.25	0.1875	0.1875	0.1875
		Max value	0.25	0.25	0.25	0.1875	0.1875	0.1875
	LB	Mode value	1.5	1.5	1	1.5	1.5	1.5
		Max value	1.5	1.5	1	2	1.5	1.5
Co^2+^	HMM	Mode value	0.75	0.75	0.75	0.094	0.094	0.094
		Max value	1	0.75	0.75	0.1875	0.1875	0.094
	LB	Mode value	3	3	3	3	1	1
		Max value	3	3	3	3	1.5	1.5

^*a*^ LB, nutrient-rich medium; HMM, minimal medium; and n.d., not determined.

^*b*^ BWΔ-C and BLΔ-C express CepA, BWΔ-p and BLΔ-p with the empty vector, BW-p and BL-p with a working AcrAB system. Tests are conducted in four replicates, with the mode value (the value that appears the most) and maximum value shown.

As expected, for all bacteria/metal pairs, the MIC values observed for cells grown in HMM are lower than in LB, with the differences ranging from 3-fold (BLΔ-C; Zn^2+^) to 10-fold (BLΔ-C; Co^2+^). Except for Zn^2+^, BW strains are more resistant to the tested metal ions than BL strains, similar to the observation with biocides and dyes. Fe^2+^ and Mn^2+^ in HMM could not be tested since the HEPES buffer in this medium can oxidize these ions (32). Overall, no significant difference in resistance levels was observed, irrespective of whether strains expressed CepA. It is noteworthy that, unlike drug efflux pumps, the expression of metal transporters is unlikely to result in a fold change in MIC. The resistance conferred by metal transporters is typically determined using other methods, such as growth curve analysis (33, 34).

### CepA increased the tolerance of *E. coli* against certain metals, but not against biocides and dyes

**Fig 2:**
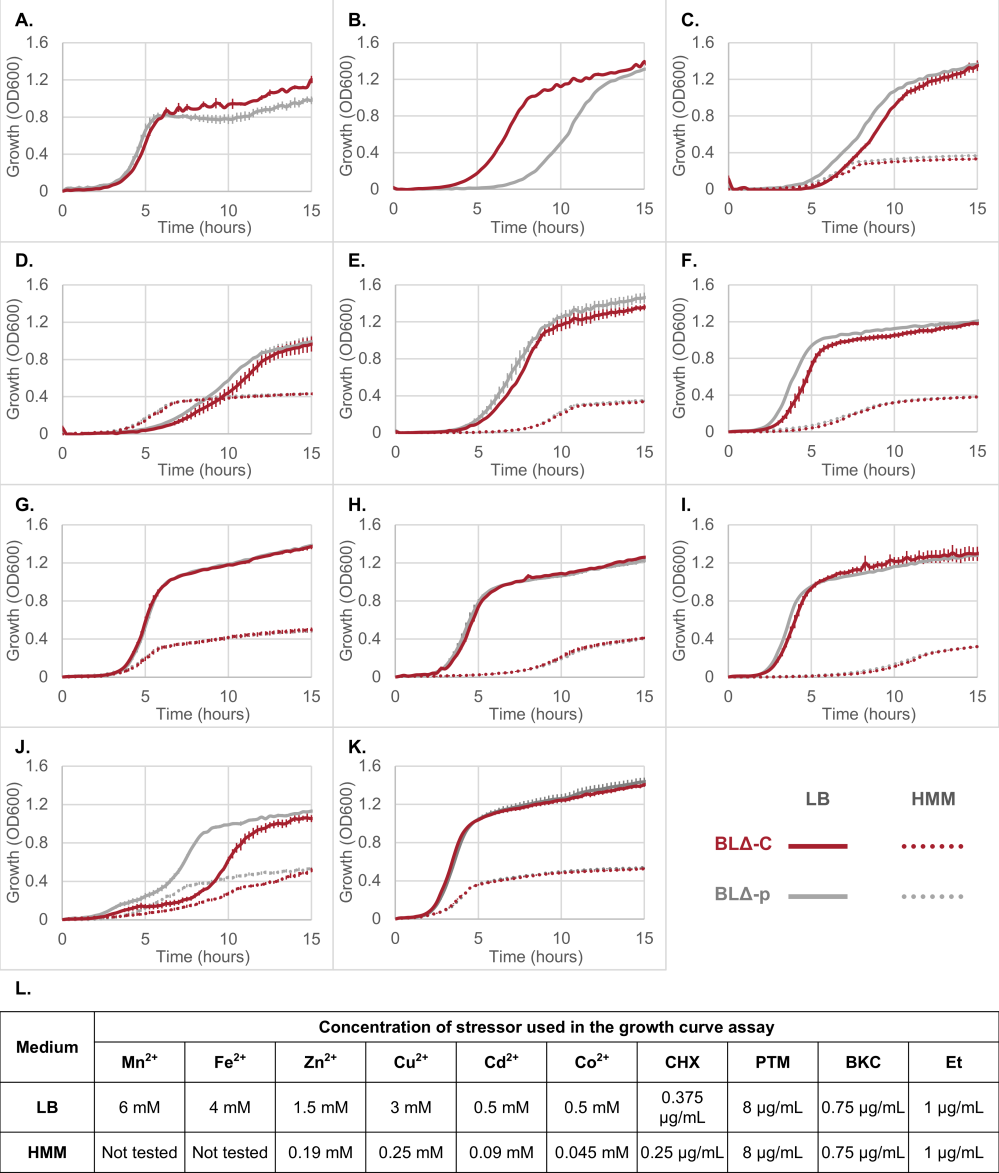
Growth of BLΔ-C and BLΔ-p cells in LB (nutrient rich) and HMM (minimal nutrition) media with different treatments. Bacteria were grown in (**A**) Mn^2+^, (**B**) Fe^2+^, (**C**) Zn^2+^, (**D**) Cu^2+^, (**E**) Cd^2+^, (**F**) Co^2+^, (**G**) chlorhexidine (CHX), (**H**) pentamidine (PTM), (**I**) benzalkonium (BKC), (**J**) ethidium (Et), or (**K**) left untreated. (**L**) Concentrations of stressor supplemented to LB and HMM media. The error bars represent the standard error of the mean of four replicates.

To further evaluate the impact of CepA on the tolerance of *E. coli* against cationic agents, the growth of BLΔ-p and BLΔ-C cells in LB and HMM media was monitored. Strains were grown with and without one of the test agents, such as chlorhexidine, benzalkonium, pentamidine, ethidium, Fe^2+^, Mn^2+^, Cu^2+^, Zn^2+^, Co^2+^, or Cd^2+^. The concentration used was guided by the previous MIC result (Table 2); for each agent, a concentration no higher than 75% MIC value while still maintaining a reasonable growth rate was chosen. The metal concentrations used for the HMM minimal medium (0.045–0.25 mM) have been observed in environmental samples polluted with heavy metals (35, 36). Meanwhile, the metal concentrations used for the nutrient-rich LB medium (0.5–6 mM) were higher to compensate for the reduction of metal toxicity, as mentioned in the previous section. These growth curves are presented in Fig. 2.

The growth curves of untreated cultures showed no difference irrespective of media or presence of CepA (Fig. 2K), indicating that the expression of CepA does not have a notable fitness cost under these conditions. A similar observation could be made with chlorhexidine, benzalkonium, and pentamidine treatments in both LB and HMM (Fig. 2G through I). However, ethidium treatment did produce distinct growth phenotypes (Fig. 2J), as biphasic growth curves were observed in both media, with the growth of BLΔ-C being heavily compromised compared to that of the BLΔ-p strain. This biphasic characteristic was more prominent in LB cultures.

No significant growth alternation was observed when cells were grown with Cu^2+^, Zn^2+^, Co^2+^, or Cd^2+^ treatment in both media. The BLΔ-p strain performed slightly better than BLΔ-C under these conditions, which might result from a fitness cost of the high CepA expression level (Fig. 2C through F). Fe^2+^ and Mn^2+^ treatments were only conducted in LB for the reason mentioned prior, and interestingly, BLΔ-p cells exhibited a noticeable growth impairment compared to BLΔ-C in these two treatments (Fig. 2A and B). A growth impairment is more significant in the Fe^2+^ treatment, with a 3-hour growth delay of BLΔ-p cells compared to BLΔ-C (Fig. 2A). Under Mn^2+^ treatment conditions, the cell density in the stationary growth phase is decreased in BLΔ-p culture (Fig. 2A).

These data suggest that CepA does not contribute to the biocide and dye resistance of BL21(DE3) ΔAcrAB strains but plays a role in the tolerance of the strains under iron and manganese stress.

### CepA presence does not influence ethidium transport in *E. coli*

**Fig 3:**
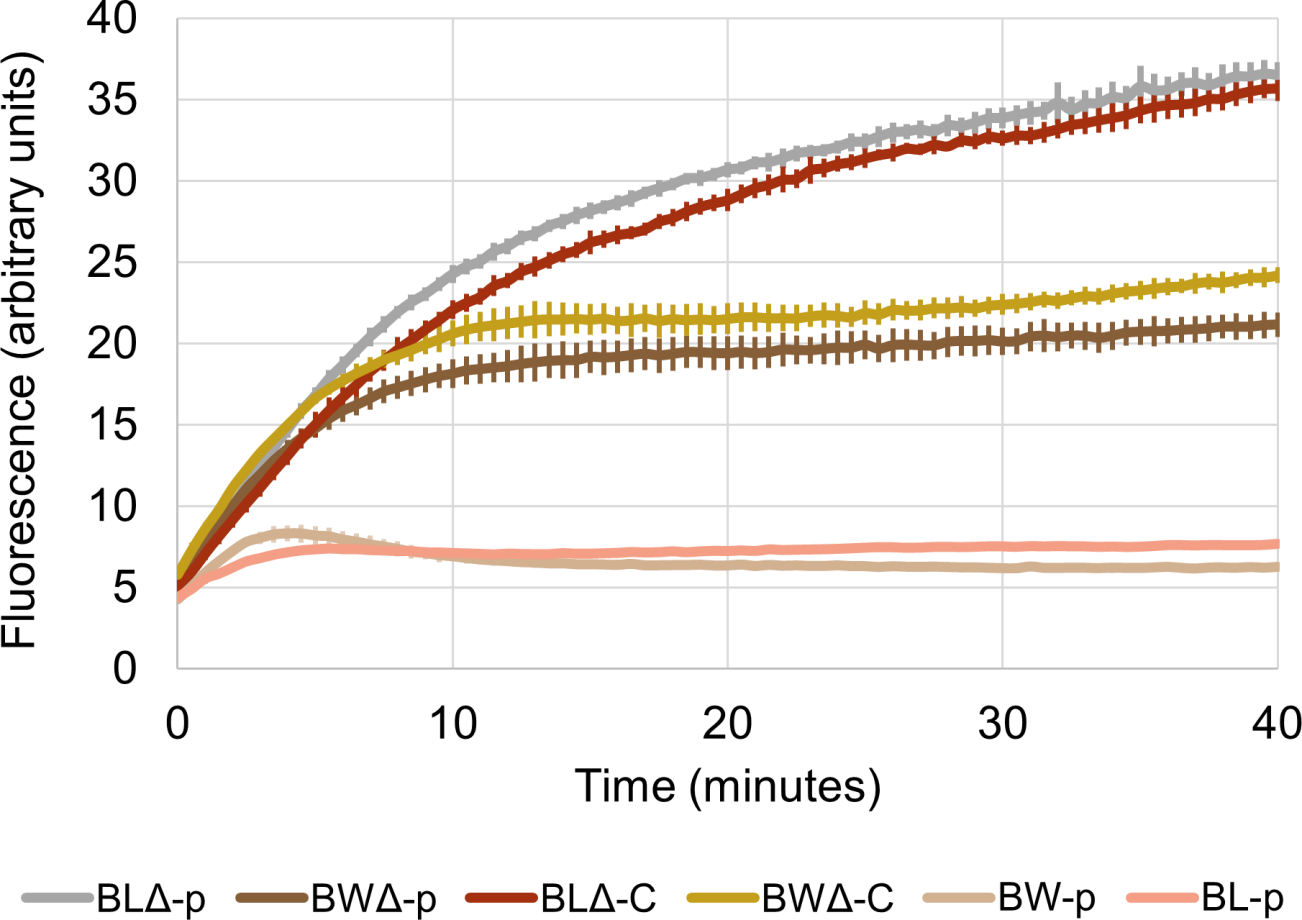
Ethidium accumulation curves of cultures of BW-p, BWΔ-p, BWΔ-C, BL-p, BLΔ-p, and BLΔ-C strains. BWΔ-C and BLΔ-C have CepA expressed, BWΔ-p and BLΔ-p, the empty vector, and BW-p and BL-p, a working AcrAB-TolC system. The error bars represent the standard error of the mean of four replicates.

To verify if CepA can extrude ethidium, an ethidium accumulation assay was conducted with BW-p, BWΔ-p, BWΔ-C, BL-p, BLΔ-p, and BLΔ-C cells. If ethidium efflux is active, then less intracellular ethidium can accumulate in cells carrying CepA, resulting in lower fluorescence. Thus, cells were exposed to ethidium bromide and energized with sodium pyruvate to initiate dye transportation; the fluorescent intensity of intracellular ethidium bromide was monitored (Fig. 3).

The BLΔ-p and BLΔ-C cells shared a similar accumulation pattern as fluorescence gradually increased over the tested period. The accumulation pattern of BWΔ-p and BWΔ-C cells was also similar to each other, with an increase in fluorescence intensity in the first 10 minutes to a level that remained stable for 20 minutes, which is approximately 50% lower than that of the BLΔ background. In contrast, BL-p and BW-p cells displayed low ethidium accumulation that peaked at 90% lower than the BLΔ background. BL-p and BW-p cells possess an intact AcrAB system and were included in this test as a control.

It could be seen that strains from the BL background exhibited a higher transport activity than their counterparts from the BW background, regardless of the presence of either the CepA or AcrAB system. These data are consistent with the MIC test, in which cells with the BW background exhibited higher MIC levels than those with the BL background for most biocides and dyes. These results suggest that BW (*E. coli* BW25113) has a greater intrinsic efflux-mediated drug resistance compared to BL [*E. coli* BL21(DE3)]. However, no differences in efflux activity between the CepA-expressed strains and strains with the empty vector were observed, which indicates that this protein does not transport ethidium in these *E. coli* strains. Therefore, the difference in the growth curve observed in the ethidium treatment (Fig. 2J) did not result from CepA-mediated efflux activity. Since ethidium is a common substrate for the majority of antimicrobial efflux pumps (29), the inability to transport this compound discourages the possibility of CepA being a biocide efflux pump.

### CepA presence resulted in decreased accumulation of certain metals in *E. coli*

**Fig 4:**
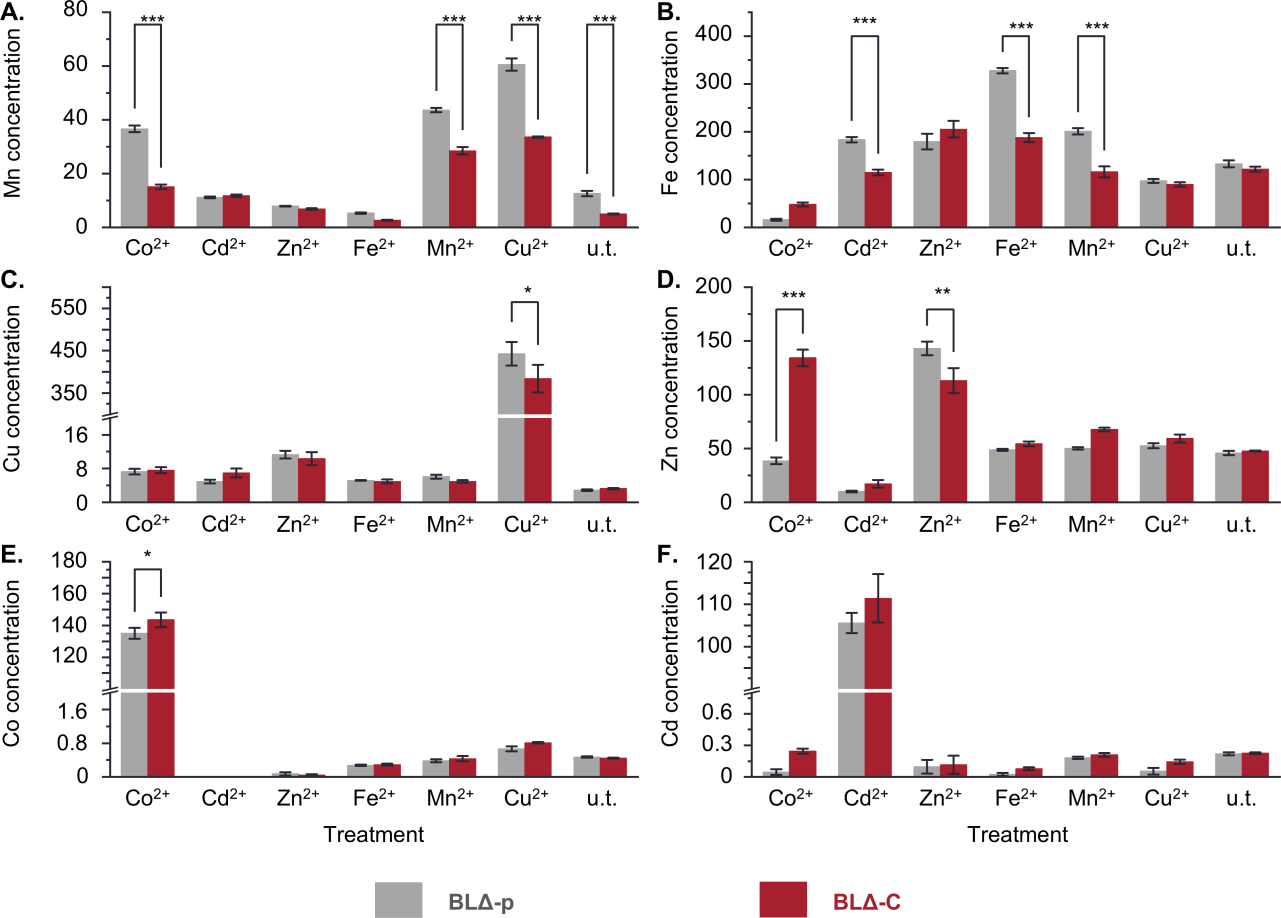
The intracellular concentration of metals in BLΔ-C and BLΔ-p cells in LB medium with different treatments. Bacteria were grown in 0.5 mM Co^2+^, 0.5 mM Cd^2+^, 1.5 mM Zn^2+^, 3 mM Fe^2+^, 4 mM Mn^2+^, or 2.5 mM Cu^2+^ or left untreated (u.t.). The concentration (µg/mL) of (**A**) manganese, (**B**) iron, (**C**) copper, (**D**) zinc, (**E**) cobalt, and (**F**) cadmium was determined using ICP-MS. The error bars represent the standard error of the mean of four replicates. Asterisks indicate significantly different pairs with *P ≤ 0.05, **P ≤ 0.01, or ***P ≤ 0.001.

An inductively coupled plasma mass spectrometry (ICP-MS) assay was conducted to compare metal accumulation between BLΔ-p and BLΔ-C strains. Bacteria were grown in LB supplemented with a sub-inhibitory concentration of each metal ion (Fe^2+^, Mn^2+^, Cu^2+^, Zn^2+^, Co^2+^, and Cd^2+^). Cells were then harvested, and the intracellular concentration of the six metals was determined using an ICP-MS assay (Fig. 4).

The BLΔ-C strain accumulated significantly less iron (Fe^2+^) and manganese (Mn^2+^) than the BLΔ-p strain in both treated and untreated conditions (Fig. 4A and B). The BLΔ-C strain also accumulated less copper (Cu^2+^) and zinc (Zn^2+^) than the strain lacking CepA BLΔ-p, but only when treated with the respective metal ion (Fig. 4C and D). In contrast, the concentrations of cobalt (Co^2+^) and cadmium (Cd^2+^) were largely similar between BLΔ-p and BLΔ-C strains in all treatments (Fig. 4E and F). Interestingly, cells expressing CepA accumulated less zinc in the Zn^2+^ treatment, but the reverse was true for other treatments, especially Co^2+^ (Fig. 4D).

These data suggest that cells carrying the CepA protein can efficiently extrude Fe^2+^ and Mn^2+^ even at a low concentration. CepA may also be involved in the extrusion of Cu^2+^ and Zn^2+^ to a lesser degree, albeit when the concentration is sufficiently high. However, the protein is likely not capable of exporting Co^2+^ and Cd^2+^.

### CepA does not play a role in metal-biocide interaction in *E. coli*

To evaluate a possible metal-biocide interaction facilitated by CepA and its effect on *E. coli* survival, BLΔ-p and BLΔ-C strains were grown in varying concentration combinations of pairs of metal-biocides. Each pair consisted of one of the metal substrates of CepA (Fe^2+^, Mn^2+^, Cu^2+^, or Zn^2+^) and one of the four biocides (benzalkonium, chlorhexidine, pentamidine, or ethidium). These metal-biocide interactions are summarized in Table 3.

**TABLE 3:** Checkerboard assay of metal-biocide interactions

Biocide^*b*^	ΣFIC^*a*^(max fold change in biocide MIC) (max fold change in metal MIC)							
	Fe^2+^		Mn^2+^		Cu^2+^		Zn^2+^	
	BLΔ-p	BLΔ-C	BLΔ-p	BLΔ-C	BLΔ-p	BLΔ-C	BLΔ-p	BLΔ-C
BKC	1.5 (1) (1)	1.5 (1) (1)	1.5 (1) (1)	1.5 (1) (1)	1.5 (1) (1)	1 (0.5) (0.5)	1 (0.5) (1)	0.75 (0.5) (0.25)
CHX	2.5 (2) (1)	2.5 (2) (1)	1.5 (1) (1)	1.5 (1) (1)	1.5 (1) (1)	1.5 (1) (1)	1.5 (1) (1)	1.5 (1) (1)
Et	2.5 (2) (1)	2.5 (2) (1)	0.625 (0.5) (0.125)	1 (0.5) (0.5)	1.5 (1) (1)	1.5 (1) (1)	1.5 (1) (1)	2.5 (2) (1)
PTM	8.5 (8) (1)	8.5 (8) (1)	2.5 (2) (1)	2.5 (2) (1)	4.5 (4) (1)	4.5 (4) (1)	2.5 (2) (1)	2.5 (2) (1)

^*a*^ The metal-biocide interaction is represented by the FIC value; see Materials and Methods. For a metal-biocide pair, an FIC ≤ 0.5 indicates a synergy, 0.5 < ΣFIC ≤ 2 indicates additivity or no interaction, and ΣFIC > 2 indicates antagonism. Experiments were done in triplicate, with the mode value shown.

^*b*^ BKC, benzalkonium; CHX, chlorhexidine; Et, ethidium; and PTM, pentamidine.

In both strains, antagonism was observed between Fe^2+^ and all biocides except for benzalkonium, and between pentamidine and all metals, with FIC values ranging from 2.5 to 8.5 (Table 3). Interestingly, in those cases, the biocide MIC increased in the presence of metal ions, but the MIC of metal ions remained constant, which indicates a one-way interaction from metals to biocides. The strongest interaction was observed in the pentamidine-iron pair, with an eightfold change in the pentamidine MIC when the concentration of Fe^2+^ reached half MIC. Other metal-biocide pairs mostly displayed no additivity or no interaction. However, no significant difference in FIC values was observed between BLΔ-p and BLΔ-C in most tested pairs.

These data suggest that the presence of certain metals can increase the tolerance of *E. coli* to biocides. However, no noticeable contribution of CepA to that phenomenon was observed.

## DISCUSSION

CepA from *K. pneumoniae* is a protein that belongs to the CDF metal transporter family; however, it has also been previously reported to be associated with chlorhexidine resistance and may act as a cation efflux pump in *K. pneumoniae* (16). This study has investigated the substrate profile of CepA and clarified its contribution to metal and biocide resistance.

Phylogenetic data indicated that CepA shares high identity and evolutionary closeness to FieF from *E. coli*. Together, they form a branch in the Fe/Zn-CDF subfamily (Fig. 1) (24). FieF has been reported to export iron and zinc; it contributes to iron detoxification but has no involvement in zinc homeostasis in *E. coli* and can also bind Cd^2+^, Ni^2+^, Co^2+^, and Mn^2+^ (37, 38). This substrate profile is very similar to that of CepA observed in this study. In *E. coli* strains expressing CepA, iron and manganese seemed to be substrates of CepA, and the tolerance to these metals increased notably. Copper and zinc are also likely substrates of CepA but with less affinity, and the tolerance of CepA-expressing strains to these metals is not visibly affected. Meanwhile, no observable transport activity of Cd^2+^ and Co^2+^ could be demonstrated. The activity of *E. coli* FieF toward manganese and copper has not been previously reported, but it can be postulated based on an 89% similarity between the two proteins. The finding that CepA can efflux both iron and manganese is understandable since these two metals are chemically and structurally very similar (39). It is worth noting that the tested *E. coli* strains still possess their own metal transporter systems, including FieF and ZitB from the CDF family (Table S1), and inactivating AcrAB did not compromise the metal tolerance of the strains (Table 2). Therefore, the tests might have failed to identify a lower activity of CepA with some metals due to the background transportation. No transcriptional regulation pathway has been reported for FieF in *E. coli*. However, the transporter has a known auto-regulation mechanism for Zn^2+^, in which a metallochaperone-like feature in the cytoplasmic domain can sense intracellular zinc concentration and lock the dimer in the active conformation. The features required for auto-regulation are highly conserved across the CDF family (40). Both *K. pneumoniae* CepA and *E. coli* FieF are located in similar genomic neighborhoods, with the two-component genes *cpxARP* downstream and *pfkA* upstream (GenBank accession numbers NC_012731 and NC_000913). With the high similarities in phylogenetics and functions between *E. coli* FieF and *K. pneumoniae* CepA, we suggest that CepA should be renamed *K. pneumoniae* FieF, and this protein plays a role in metal homeostasis and iron detoxification in *Klebsiella*.

This study failed to observe any contribution of CepA to the resistance of the tested *E. coli* strains to biocides and dyes. Based on the ethidium accumulation data, it is also unlikely that CepA can transport cationic molecules. The crystal structure of *E. coli* FieF has been previously reported (41), and it is believed to be highly tuned for the transportation of small metal ions (25). Based on the FieF crystal structure and the amino acid sequence similarity between *E. coli* FieF and CepA, it is uncertain how the latter can transport large molecules like chlorhexidine. Previous literature stated that mutation of CepA has never been observed following chlorhexidine exposure, and Enterobacteriaceae strains that are hypersensitive to chlorhexidine all seem to possess a *cepA* gene (42). The data suggest that CepA is not a transporter of chlorhexidine and other cationic molecules.

However, CepA may be indirectly associated with chlorhexidine resistance via a non-efflux-mediated mechanism. The correlation between metals and antimicrobial resistance has been reported in various papers. Planktonic and biofilm growth of *E. coli* can be synergistically inhibited by a combination of metal ions (Cu^2+^ or Al^3+^) and quaternary ammonium compounds (cetylpyridinium or benzalkonium) (43). The presence of metal ions such as Cd^2+^ and Cu^2+^ can regulate the expression of Resistance-Nodulation-Division (RND) efflux pumps that can extrude biocides and antibiotics (44). Zn^2+^ and Fe^2+^ homeostasis is essential for biofilm formation, motility, resistance, and oxidative stress survivability of bacteria, including *Klebsiella* (45, 46). Cu^2+^, Ga^2+^, and Ti^2+^ can prevent biofilm formation in *P. aeruginosa*, *S. aureus,* and *E. coli* (47). These factors can impact the susceptibility of bacteria to antimicrobials, including chlorhexidine. The data from this study also showed that the tolerance of *E. coli* to biocides increases in the presence of heavy metals, suggesting a cross-resistance mechanism between the two. However, since no noticeable contribution of CepA to this interaction was detected, it is unlikely that it plays a direct role in the biocide resistance pathway. Nevertheless, since this study used *K. pneumoniae* protein CepA expressed in the heterologous *E. coli* host, it is possible that CepA participates in a pathway that contributes to the innate chlorhexidine resistance in *K. pneumoniae* that does not exist in *E. coli*. This remains unconfirmed since there has been no further study on the function and substrate profile of CepA other than the first publication (16), and previous studies on the correlation between biocide resistance and the presence of *cepA* provided mixed results (20, 22, 48–51). For instance, Abuzaid et al. (50) reported that the expression of *cepA* of *K. pneumoniae* isolates is positively correlated with the MIC of chlorhexidine against them, while Naparstek et al. (51) reported that the reduced susceptibility of *K. pneumoniae* to chlorhexidine is independent of the expression of *cepA*.

Efflux-mediated chlorhexidine resistance has been observed across bacterial lineages. The involved pumps all belong to established families/superfamilies, such as AcrAB (Enterobacteriaceae) of the RND superfamily, AceI (*A*cine*tobacter baumannii*) of the Proteobacterial Antimicrobial Compound Efflux family, QacC (*Staphylococcus aureus*) of the Small Multidrug Resistance family, or QacA (*S. aureus*) of the Major Facilitator superfamily (13, 52). Metal efflux pumps are also diverse and come from different families/superfamilies, such as the P-type ATPase family (53), the CorA family (54), and the CDF family, of which CepA is a member. No proteins are known to be associated with the transport and resistance of both metals and biocides, although cross-resistance between the two is not uncommon due to the co-occurrence of metal efflux pumps with biocide efflux pumps on mobile genetic elements (55, 56). Previous publications on CepA (16, 20, 21, 48) suggest that CepA from *K. pneumoniae* is the first known protein to export both biocide and metal. This study evaluated its substrate profile, albeit in the heterologous host *E. coli*. We confirmed the metal transport activity of CepA, which is typical for a CDF protein, but failed to observe any association of this protein with chlorhexidine resistance. Further studies need to investigate the function and substrate profile of CepA in the native host, *K. pneumoniae*, before establishing the association of this protein with chlorhexidine and biocide resistance, as well as its role in *K. pneumoniae* metal homeostasis. Further examination of other CDF proteins is desirable to gain insight into the possible role they play in the antimicrobial resistance of bacteria.

## MATERIALS AND METHODS

### Bacterial strains

*E. coli* DH5α and *E. coli* BL21(DE3) were purchased from Invitrogen (USA), *E. coli* BW25113 and *E. coli* BW25113 Δ*acrAB* were a generous gift from Associate Professor Rietie Venter (UniSA, Australia) (57), and *E. coli* DH5α Δ*acrAB* and *E. coli* BL21(DE3) Δ*acrAB* strains were generated in our laboratory using the bacteriophage λ Red recombinase-based gene inactivation method (58). Other strains were generated in this study. A list of *E. coli* strains and their features is included in Table S2.

All bacteria were routinely maintained in LB (tryptone, 10 g/L; yeast extract, 5 g/L; and NaCl, 5 g/L in MilliQ water) and LB agar (LB broth with 16 g/L agar); 100 µg/mL of ampicillin was supplemented to maintain plasmid presence where appropriate.

### Phylogenetic analysis of the CepA protein

The *cepA* nucleotide sequence (NCBI GenBank accession no. AB073019) (16) was subjected to an NCBI Standard Nucleotide BLAST search (59) to verify the presence of the gene in *K. pneumoniae* genomes. Amino acid sequences of different CDF proteins were also retrieved from NCBI GenBank (Table S1). All amino acid sequences were aligned using the Clustal Omega program on the EMBL-EBI website (60). The multiple sequence alignment was visualized using the ESPript 3.0 online tool (61). The multiple sequence alignment was used to construct a phylogenetic tree using IQ-TREE 2.2.0 for Windows (62) using the automatic model selection for nucleotide substitution, default heuristic search options, and ultrafast bootstrapping with 10,000 replicates. A visualization of the phylogenetic tree was generated using the iTOL v6 online tool (63). The similarity and relationship between CepA and other CDF proteins were evaluated.

### Cloning the CepA protein into bacterial strains

The amino acid sequence of CepA (NCBI GenBank accession no. AB073019) was used to design and generate an optimized gene sequence using the GenSmart Codon Optimization online tool (GenScript); the gene fragment was synthesized by Integrated DNA Technologies. Eight histidine codons were added to the sequence before the stop codon. A leader sequence of T7 phage gen 10 was inserted seven nucleotides upstream of the gene start codon, and *Eco*RI and *Sac*I restriction sites were inserted at the 5′ and 3′ ends of the sequence, respectively (Fig. S3). The fragment was digested with *Eco*RI and *Sac*I and ligated into a similarly digested pBlueScript II SK (+) vector (Agilent), putting it under the regulation of the T7 promoter. The ligated product, pBS-CepA, was chemically transformed into *E. coli* DH5α. Transformants were plated on LB agar with ampicillin 100 µg/mL, and single colonies were picked and grown overnight in LB with ampicillin. Plasmids were isolated from overnight cultures using the ISOLATE II Plasmid Mini Kit (Bioline). The sequence of the inserted gene was verified by whole-plasmid sequencing. The strain was called DH5α (pBS-CepA). The plasmid was also transformed into *E. coli* DH5α Δ*acrAB*, *E. coli* BL21(DE3) Δ*acrAB*, and *E. coli* BW25113 Δ*acrAB* to create strains *E. coli* DH5α Δ*acrAB* (pBS-CepA), *E. coli* BL21(DE3) Δ*acrAB* (pBS-CepA), and *E. coli* BW25113 Δ*acrAB* (pBS-CepA). The empty pBlueScript II SK (+) was transformed into *E. coli* DH5α Δ*acrAB*, *E. coli* BL21(DE3) Δ*acrAB*, and *E. coli* BW25113 Δ*acrAB* to create strains *E. coli* DH5α Δ*acrAB* (pBS), *E. coli* BL21(DE3) Δ*acrAB* (pBS), and *E. coli* BW25113 Δ*acrAB* (pBS).

### Membrane protein isolation and Western blot analysis

Membrane protein samples were prepared using a modification of the protocol from reference 64. All bacterial strains were inoculated in LB. Strains BL21(DE3) Δ*acrAB* (pBS) and BL21(DE3) Δ*acrAB* (pBS-CepA) were grown with aas inspected in nd without the addition of IPTG 100 µM to induce the expression of CepA. Cells were harvested at an OD_600_ of 1 and washed with ice-cold TBS buffer (Tris 20 mM [pH 7.5] and NaCl 150 mM in Milli-Q water) with centrifugation at 4,000 *g* at 4°C for 5 minutes after both steps. Washed cell pellets were resuspended in 30 mL of Crushing Buffer (glycerol 10% in TBS buffer) and passed twice through a pre-chilled high-pressure cell disruptor (Constant Systems) at 30,000 psi. The suspension was centrifuged at 4,000 *g* at 4°C for 1 hour to remove unbroken cells and debris. The supernatant was collected and centrifuged at 126,000 *g* at 4°C for 2 hours to pellet membrane fragments. The final supernatant was discarded, and 750 µL of the Membrane Resuspension Buffer (Tris-HCl 20 mM [pH 7.5], glycerol 10% [wt/vol], and DDM 2% [wt/vol] in Milli-Q water) was added to the pellet. The submerged pellets were incubated overnight in a shaking incubator at 150 rpm at 4°C to solubilize membrane proteins.

Protein concentration in all samples was quantified using the DC Protein Assay (Bio-Rad) and standardized to 4 mg/mL. SDS-PAGE of all samples was performed using the Bio-Rad Mini-PROTEAN Tetra system. For total protein visualization, the developed SDS-PAGE polyacrylamide gel was stained with Colloidal Coomassie G-250 following a published protocol (65). Another developed SDS-PAGE polyacrylamide gel was Western-blotted onto a PVDF membrane. The membrane was incubated with a rabbit anti-His-tag antibody (Rockland Immunochemicals), then with a horseradish-peroxidase-conjugated goat-anti-rabbit IgG antibody (Bio-Rad). His-tagged CepA band was visualized using Bio-Rad Clarity Western ECL Substrate under a ChemiDoc MP Imaging System (Bio-Rad).

### Minimal inhibitory concentration assay

The MIC assay for selected bacterial strains was conducted using the 96-well plate micro broth dilution method, essentially as described in a previous publication (66). The test media used for MIC assay are Mueller-Hinton Broth (MHB; Oxoid) for biocides and dyes, and LB for metal ions. For four metal ions, Cu^2+^, Zn^2+^, Cd^2+^, and Co^2+^, an additional MIC assay was conducted with a HMM (NaCl, 0.5 g/L; NH_4_Cl, 1 g/L; casamino acid, 2 g/L; glycerol, 0.4% [wt/vol]; HEPES, 20 mM; MgSO_4_, 2 mM; thiamine, 1 mM; CaCl_2_, 0.1 mM; K_2_HPO_4_ , 0.1 mM in Milli-Q water, pH to 7.0 with NaOH, filter sterilized).

Cultures of *E. coli* strains were grown overnight in the test medium with ampicillin (100 µg/mL). Overnight cultures were subcultured to an OD_600_ value of 0.3 with 100 µg/mL ampicillin. The subcultures were diluted to 1/100 concentration in the test medium without antibiotics. From that, 10 µL was inoculated into wells containing 90 µL of the test medium with a concentration range of substrate. The test substrates include biocides (benzalkonium chloride, chlorhexidine hydrochloride, pentamidine isethionate, and methyl viologen; Sigma), dyes (ethidium bromide, acridine orange, acriflavine, proflavine, pyronin Y, methyl green, crystal violet, and methylene blue; Sigma), and metal ions (Fe^2+^ from FeSO_4_, Mn^2+^ from MnSO_4_, Cu^2+^ from CuSO_4_, Zn^2+^ from ZnSO_4_, Cd^2+^ from CdSO_4_, and Co^2+^ from CoCl_2_; Sigma). The plates were incubated at 37°C with light shaking and were checked after 48 hours.

### Growth curve assay

Cultures of selected *E. coli* strains were grown overnight in LB with 100 µg/mL ampicillin. Overnight cultures were subcultured to an OD_600_ value of 0.3 in LB with 100 µg/mL ampicillin. The subcultures were diluted to 1/10 concentration in LB without antibiotics. From that, 20 µL was inoculated into wells containing 180 µL of MHB with or without the supplement of a test substrate. The plate was incubated at 37°C with shaking in a SPECTROstar Nano spectrophotometer (BMG Labtech) for 18 hours, and the OD_600_ value of all wells was monitored every 15 minutes. The test substrates include biocides (benzalkonium chloride, chlorhexidine hydrochloride, and pentamidine isethionate; Sigma), dyes (ethidium bromide; Sigma), and metal ions (Fe^2+^, Mn^2+^, Cu^2+^, Zn^2+^, Cd^2+^, and Co^2+^; Sigma). For four metals, Cu^2+^, Zn^2+^, Cd^2+^, and Co^2+^, an additional growth curve assay was conducted with HMM medium.

### Ethidium accumulation assay

Cultures of selected *E. coli* strains were grown overnight in LB with 100 µg/mL ampicillin. The overnight culture was subcultured in LB with 100 µg/mL ampicillin and grown to an OD_600_ value of 1.5. Three milliliters of subculture was pelleted and washed three times in HEPES buffer (20 mM, pH 7) by centrifugation before resuspension in 1 mL of HEPES buffer. From that, 100 µL of the resuspension was loaded into each well of a 96-well black plate, followed by 100 µL of a solution containing 400 mM sodium pyruvate and 20 mM ethidium bromide. The plate was incubated at 37°C with shaking in a CLARIOstar spectrophotometer (BMG Labtech), and the fluorescent intensity of all wells was monitored every 30 seconds with an excitation and emission wavelength of 526 and 605 nm, respectively.

### Cellular metal content analysis

Cultures of selected *E. coli* strains were grown overnight in LB with 100 µg/mL ampicillin. The overnight culture was subcultured to an OD_600_ value of 0.7 in LB with and without the addition of the six metal ions mentioned above. Cells were harvested and washed three times with EDTA saline (NaCl 0.9% and EDTA 5 mM in Milli-Q water) and then three times with saline (NaCl, 0.9%) using centrifugation. Bacterial pellets were desiccated overnight at 95°C and digested in full-strength HNO_3_ at 90°C for 1 hour. Samples were diluted to 1/10 concentration with Milli-Q water and analyzed by ICP-MS on an Agilent Solution 8900 QQQ ICP-MS (Flinders Analytical, Flinders University).

### Checkerboard assay

To analyze the interaction between a metal and a biocide, the checkerboard assay was performed following a modification of the protocol from (67). Cultures of *E. coli* strains were grown overnight in the test medium with 100 µg/mL ampicillin. Overnight cultures were subcultured to an OD_600_ value of 0.3 with 100 µg/mL ampicillin. A 96-well plate was prepared with a row-wise twofold serial dilution of the metal and a column-wise twofold serial dilution of the biocide in LB medium at 90 µL per well. All wells were then inoculated with 10 µL of the prepared *E. coli* culture to a cell density of approximately 5 × 10^6^ CFU/mL. The plate was incubated at 120 rpm 37°C for up to 48 hours. The MICs of the biocide/metal alone and of all isoeffective combinations were determined as the lowest concentrations that inhibit the visible growth of bacteria in the well.

For all wells with associated MIC values, the sum of fractional inhibitory concentration (ΣFIC) was calculated for each well with the equation:


$$\Sigma FIC\;=\;{FIC}_{A}\;+\;{FIC}_{B}\;=\;\left(C_{A}/{MIC}_{A}\right)\;+\;\left(C_{B}/{MIC}_{B}\right)$$

where MIC_*A*_ and MIC_*B*_ are the MICs of the biocide and metal alone, respectively, and *C*_*A*_ and *C*_*B*_ are the concentrations of both compounds in combination, respectively, in all wells corresponding to a MIC. A ΣFIC ≤ 0.5 indicates synergy between the metal and the biocide, 0.5 < ΣFIC ≤ 2 indicates additivity or no interaction, and ΣFIC > 2 indicates antagonism between the two compounds (68).

### Data analysis

All experiments were conducted in four replicates, consisting of two biological and two technical replicates. Data were analyzed using OriginPro 2023 (OriginLab). Statistical significance was determined by one-way analysis of variance and Tukey’s test, in which a *P*-value smaller than 0.05 was considered to be statistically significant.

## SUPPLEMENTAL MATERIAL

Supplemental material msphere.00512-25-s0001.pdf Fig. S1–S6 and Tables S1 and S2.

ASM does not own the copyrights to Supplemental Material that may be linked to, or accessed through, an article. The authors have granted ASM a non-exclusive, world-wide license to publish the Supplemental Material files. Please contact the corresponding author directly for reuse.
