# Supplementary material for: Is CepA from Klebsiella pneumoniae a biocide pump? Evidence suggests a metal efflux function
Source: mSphere. 2025 Nov 17;10(12):e00512-25. doi: 10.1128/msphere.00512-25 (PMC12724285; doi:10.1128/msphere.00512-25)
Supplement: Supplemental material — Fig. S1–S6 and Tables S1 and S2. [file msphere.00512-25-s0001.pdf]

Is CepA from *Klebsiella pneumoniae* a Biocide Pump? Evidence Suggests Metal Efflux Function

Le Phung Hien and Melissa H. Brown

SUPPLEMENTARY DATA

Figure S1. Alignment of *Klebsiella pnemoniae* CepA with selected proteins from the CDF family. 5

Figure S2. Alignment of the AlphaFold-predicted structure of *Klebsiella pnemoniae* CepA with the crystal structure of *E. coli* FieF 6

Figure S3. DNA sequence of the codon-optimized *cepA* gene 7

Figure S4. Alignment of the original sequence of *cepA* taken from *Klebsiella pneumoniae* and the codon-optimized sequence of *cepA* modified for the expression in *E. coli* with an 8xHis-tag sequence included. 8

Figure S5. Detection of CepA by a Western Blot of 45 µg of total membrane fraction isolated from different bacterial strains 9

Figure S6. Commasine-stained SDS-PAGE gel of 45 µg of total membrane fraction samples.. 10

Table S1. List of CDF protein sequences used for multiple alignments and phylogenetic tree analysis in this study 2

Table S2. List of *E. coli* strains used in this study and their notable features 11

Table S1. List of CDF protein sequences used for multiple alignments and phylogenetic tree analysis in this study

Bacterial group	Protein ID	Main function	GenBank Accession No.	Number of residues	Query coverage[a] (%)	Identities[b] (%)
Klebsiella pneumoniae	CepA	Putative chlorhexidine transporter	AB073019.1	300	100	100
Salmonella	FieF	Putative Fe^{2+} transporter	WP_001541240.1	300	99	88.22
Escherichia coli	FieF	Fe^{2+} transporter	WP_096967255.1	300	97	89
Shewanella oneidensis	FieF	Putative Fe^{2+} transporter	WP_011074103.1	296	96	49.31
Maricaulis maris	Unnamed CDF	Unknown function	WP_011642059.1	323	98	39.27
Thermus thermophilus	CzrB	Putative Zn^{2+} transporter	WP_011173120.1	291	87	34.46
Cupriavidus metallidurans	FieF	Putative Fe^{2+} transporter	WP_029306879.1	321	85	27.13
Streptococcus pneumoniae	MntE	Mn^{2+} transporter	WP_000813913.1	394	96	27.15
Bacillus cereus	CzcD	Zn^{2+} transporter	AAP09225.1	296	88	25.37
Yersinia pseudotuberculosis	ZitB	Putative Zn^{2+} transporter	WP_002210743.1	312	79	27.27
Clostridium tetani	E88	Unknown function	WP_011099261.1	289	87	21.4
Magnetospirillum magnetotacticum	MamV	Magnetite nucleation	WP_236686271.1	322	81	24.9
Magnetospirillum magnetotacticum	MamM	Magnetite nucleation	WP_024080586.1	318	91	24.65
Bacillus	Unnamed CDF	Unknown function	WP_003229873.1	311	85	22.39
Pseudomonas aeruginosa	CzcD	Zn^{2+} transporter	WP_128651262.1	299	67	24.63
Staphylococcus aureus	CzrB	Zn^{2+} transporter	WP_258413469.1	326	70	20.56
Acinetobacter baumannii	CzcD	Zn^{2+} transporter	SSQ49248.1	318	75	23.28
Escherichia coli	ZitB	Zn^{2+} transporter	WP_152930928.1	313	88	24.64
Streptococcus pneumoniae	CzcD	Zn^{2+} transporter	WP_219575289.1	296	59	24.73
Cupriavidus metallidurans	CzcD	Zn^{2+} transporter	WP_035883588.1	316	75	23.01
Mycolicibacterium smegmatis	ZitA	Zn^{2+} transporter	WP_011727169.1	297	61	26.38
Cupriavidus metallidurans	DmeF	Co^{2+} transporter	WP_029306776.1	345	21	31.25
Planococcus dechangensis	MceT	Na^{+} transporter	QCB20471.1	310	56	21.98
Klebsiella pneumoniae	ZiiP	Zn^{2+} transporter	SSW79093.1	213	35	30.84
Acinetobacter baumannii	CzcE	Zn^{2+} transporter	WP_031985740.1	213	35	30.84
Streptomyces	MMT1	Unknown function	WP_011029057.1	234	u.d.	u.d.
Acinetobacter baumannii	CzcF	Zn^{2+} transporter	WP_150378257.1	298	u.d.	u.d.
Burkholderiales	PbtF	Pb^{2+} transporter	WP_011255215.1	211	u.d.	u.d.

[a, b] Coverage and identity percentage in the amino acid sequence compared to *Klebsiella* CepA (AB073019.1)
u.d. Undetermined due to low coverage and identity.

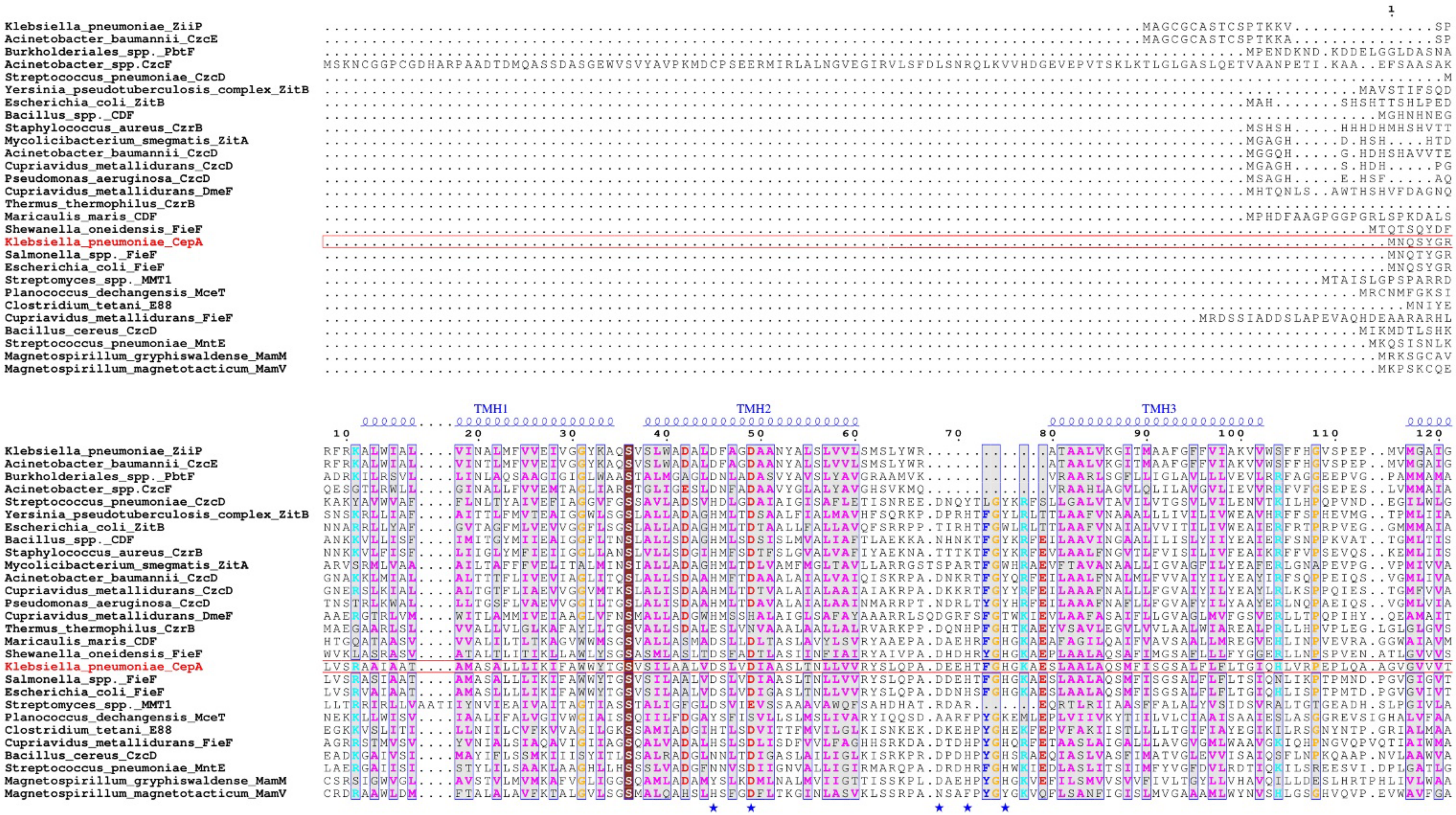

Klebsiella_pneumoniae_ZiiP
Acinetobacter_baumannii_CzcE
Burkholderiales_spp._PbtF
Acinetobacter_spp.CzcF
Streptococcus_pneumoniae_CzcD
Yersinia_pseudotuberculosis_complex_ZitB
Escherichia_coli_ZitB
Bacillus_spp._CDF
Staphylococcus_aureus_CzrB
Mycolicibacterium_smegmatis_ZitA
Acinetobacter_baumannii_CzcD
Cupriavidus_metallidurans_CzcD
Pseudomonas_aeruginosa_CzcD
Cupriavidus_metallidurans_DmeF
Thermus_thermophilus_CzrB
Maricaulis_maris_CDF
Shewanella_oneidensis_FieF
Klebsiella_pneumoniae_CepA
Salmonella_spp._FieF
Escherichia_coli_FieF
Streptomyces_spp._MMT1
Planococcus_dechangensis_MceT
Clostridium_tetani_E88
Cupriavidus_metallidurans_FieF
Bacillus_cereus_CzcD
Streptococcus_pneumoniae_MntE
Magnetospirillum_gryphiswaldense_MamM
Magnetospirillum_magnetotacticum_MamV
TMH1
TMH2
TMH3

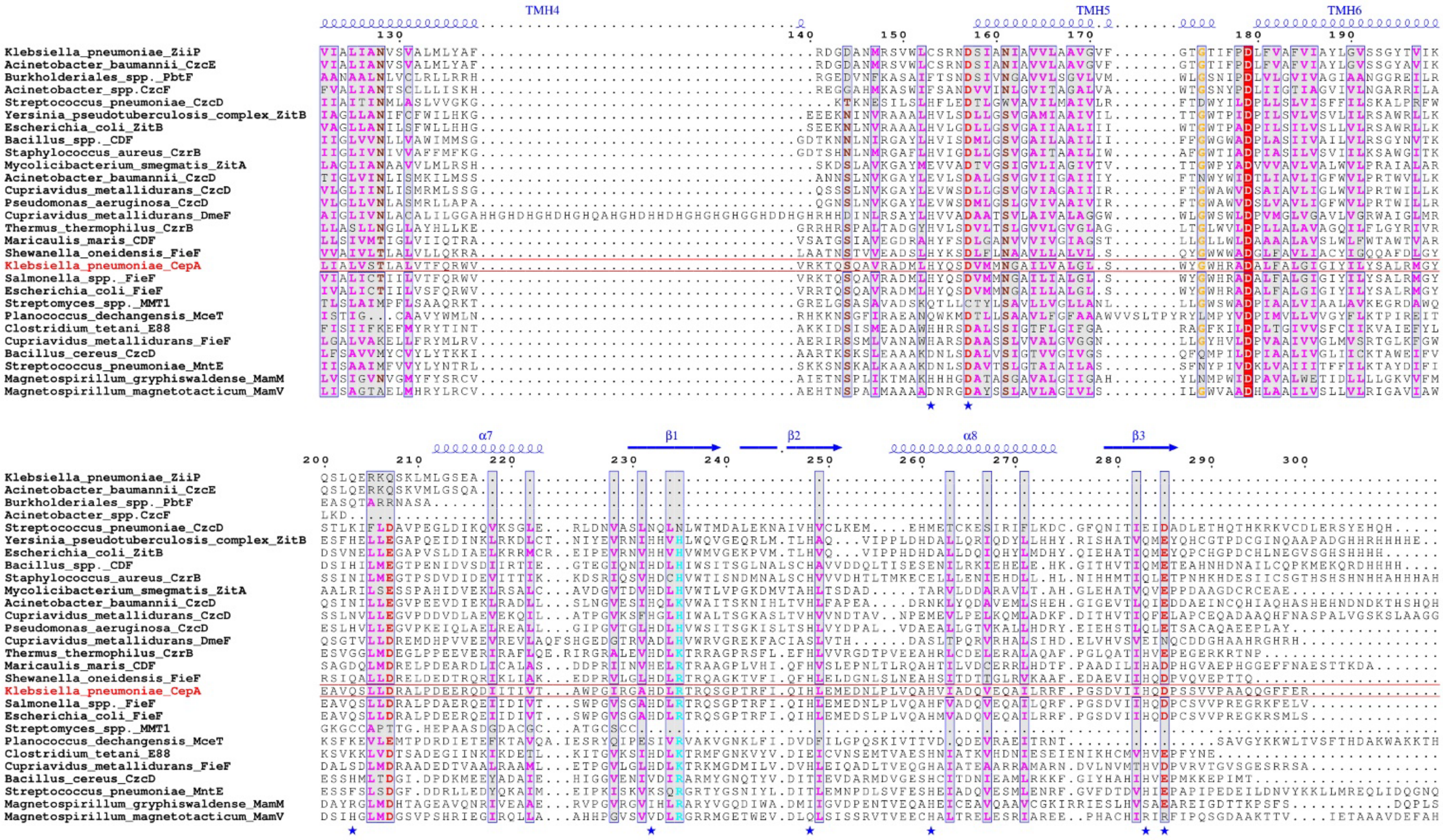
TMH4
TMH5
TMH6
α7
β1
β2
α8
β3
Klebsiella_pneumoniae_ZiiP
Acinetobacter_baumannii_CzcE
Burkholderiales_spp._PbtF
Acinetobacter_spp.CzcF
Streptococcus_pneumoniae_CzcD
Yersinia_pseudotuberculosis_complex_ZitB
Escherichia_coli_ZitB
Bacillus_spp._CDF
Staphylococcus_aureus_CzrB
Mycolicibacterium_smegmatis_ZitA
Acinetobacter_baumannii_CzcD
Cupriavidus_metallidurans_CzcD
Pseudomonas_aeruginosa_CzcD
Cupriavidus_metallidurans_DmeF
Thermus_thermophilus_CzrB
Maricaulis_maris_CDF
Shewanella_oneidensis_FieF
Klebsiella_pneumoniae_CepA
Salmonella_spp._FieF
Escherichia_coli_FieF
Streptomyces_spp._MMT1
Planococcus_dechangensis_MceT
Clostridium_tetani_E88
Cupriavidus_metallidurans_FieF
Bacillus_cereus_CzcD
Streptococcus_pneumoniae_MntE
Magnetospirillum_gryphiswaldense_MamM
Magnetospirillum_magnetotacticum_MamV

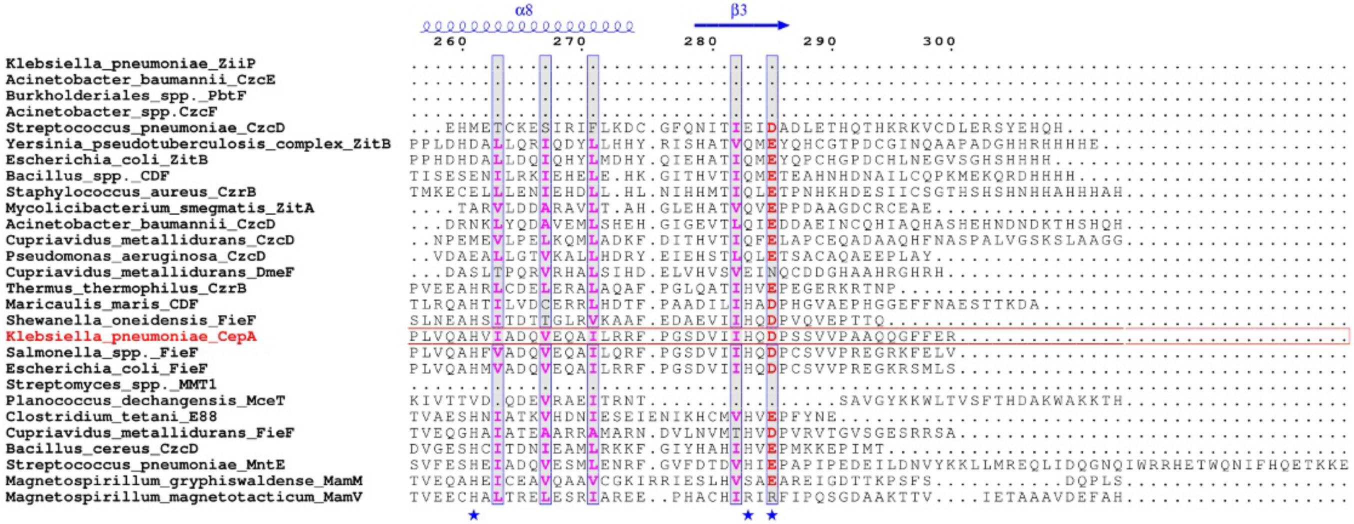

Figure S1. Alignment of *Klebsiella pnemoniae* CepA with selected proteins from the CDF family. GenkBank accession numbers of proteins are listed in Supplementary Table S1. Residue similarity was calculated considering their physicochemical properties. Columns with residue similarity greater than 70% are framed, and the residues are colored based on their physicochemical properties (cyan for polar positive, red for polar negative, brown for polar neutral, magenta for non-polar aliphatic, blue for non-polar aromatic, yellow for others). Six predicted transmembrane helices, two α-cytoplasmic helices, and three β strands are shown above the alignment. The residues important for metal ion binding and transportation of *E. coli* FieF are marked with blue stars in the bottom line.

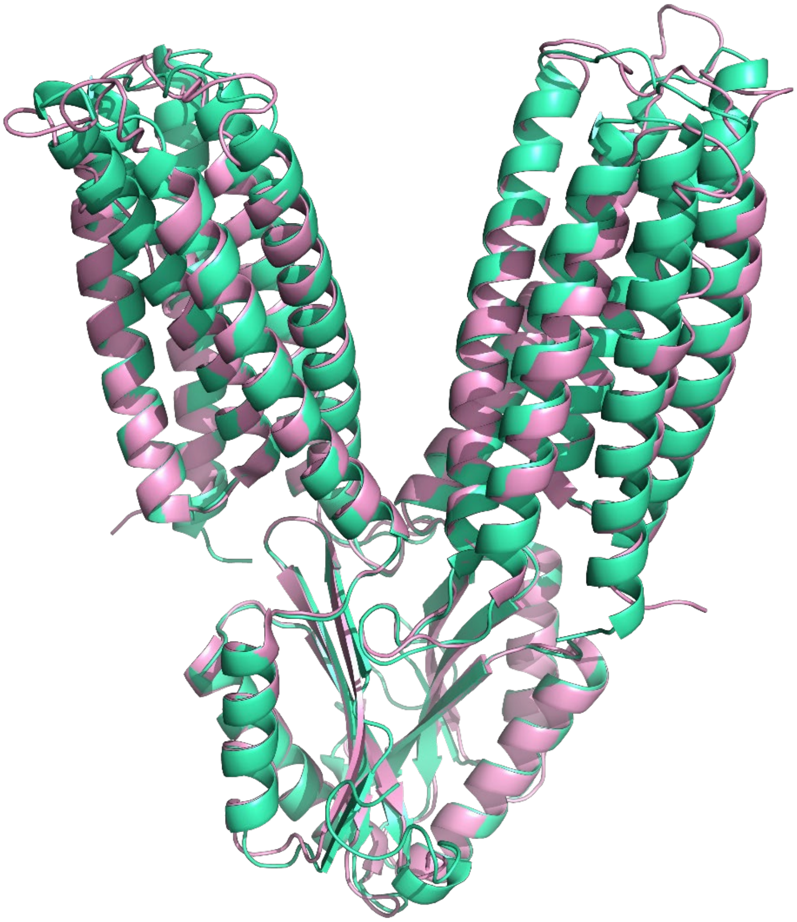

Figure S2. Alignment of the AlphaFold-predicted structure of *Klebsiella pnemoniae* CepA (Cyan) with the crystal structure of *E. coli* FieF (PDB No. 3H90, Pink), exhibiting high similarity between the two proteins (RMSD = 0.513).

```
AAAAACTCGAGAATTCTTTGTTTAACTTTAAGAAGGAGATATACATATGAATCAATCATATGGAAGGCTA     70
GTAAGTAGAGCGGCGATCGCGGCTACCGCAATGGCGAGCGCGTTACTGCTGATCAAAATTTTTGCATGGT    140
GGTATACCGGTTCTGTTTCCATTCTCGCCGCGTTGGTCGACAGCTTGGTGGACATCGCAGCCTCCCTGAC    210
CAATCTGCTTGTGGTGCGCTACTCGCTGCAACCGGCAGACGAGGAGCACACCTTCGGCCATGGTAAAGCG    280
GAAAGCCTGGCAGCCTTGGCTCAATCTATGTTTATTAGCGGCAGTGCACTTTTCCTGTTCTTGACGGGCA    350
TCCAACATCTGGTACGTCCGGAACCGCTGCAGGCGGCGGGTGTTGGTGTGGTTGTAACTCTGATTGCGCT    420
CGTCAGCACCCTGGCGCTGGTCACGTTTCAGCGTTGGGTTGTGCGGAAGACCCAGTCGCAAGCCGTTCGC    490
GCGGACATGCTGCATTATCAGTCCGATGTTATGATGAACGGCGCAATTCTAGTGGCCCTGGGCCTGAGCT    560
GGTACGGCTGGCATCGTGCCGATGCGTTGTTCGCGCTGGGCATTGGCATCTACATCTTATACTCTGCCTT    630
GCGTATGGGTTATGAGGCAGTGCAATCACTCCTGGATCGTGCGCTGCCTGATGAGGAGCGCCAGGACATC    700
ATCACCATTGTTACGGCTTGGCCGGGTATCCGTGGTGCGCACGATCTGCGTACCCGTCAAAGCGGTCCGA    770
CCCGCTTTATCCAGATACACCTGGAAATGGAAGATAACCTGCCGCTGGTGCAAGCGCATGTTATCGCGGA    840
CCAGGTTGAACAGGCGATTCTGCGCCGTTTCCCGGGTTCCGACGTGATCATTCATCAGGACCCAAGCAGC    910
GTGGTTCCGGCTGCTCAGCAGGGTTTTTCGAGCGTCATCATCACCATCACCACCATCACTAAGAGCTCA    980
AGCTTAAAAA          990
```

Figure S3. DNA sequence of the codon-optimized *cepA* gene. The highlighted nucleotides indicate the restriction sites *Eco*RI (red) and *Sac*I (magenta), the T7 phage gene 10 leader sequence (gray), and the *cepA* coding region including an 8xHis-tag sequence (purple).

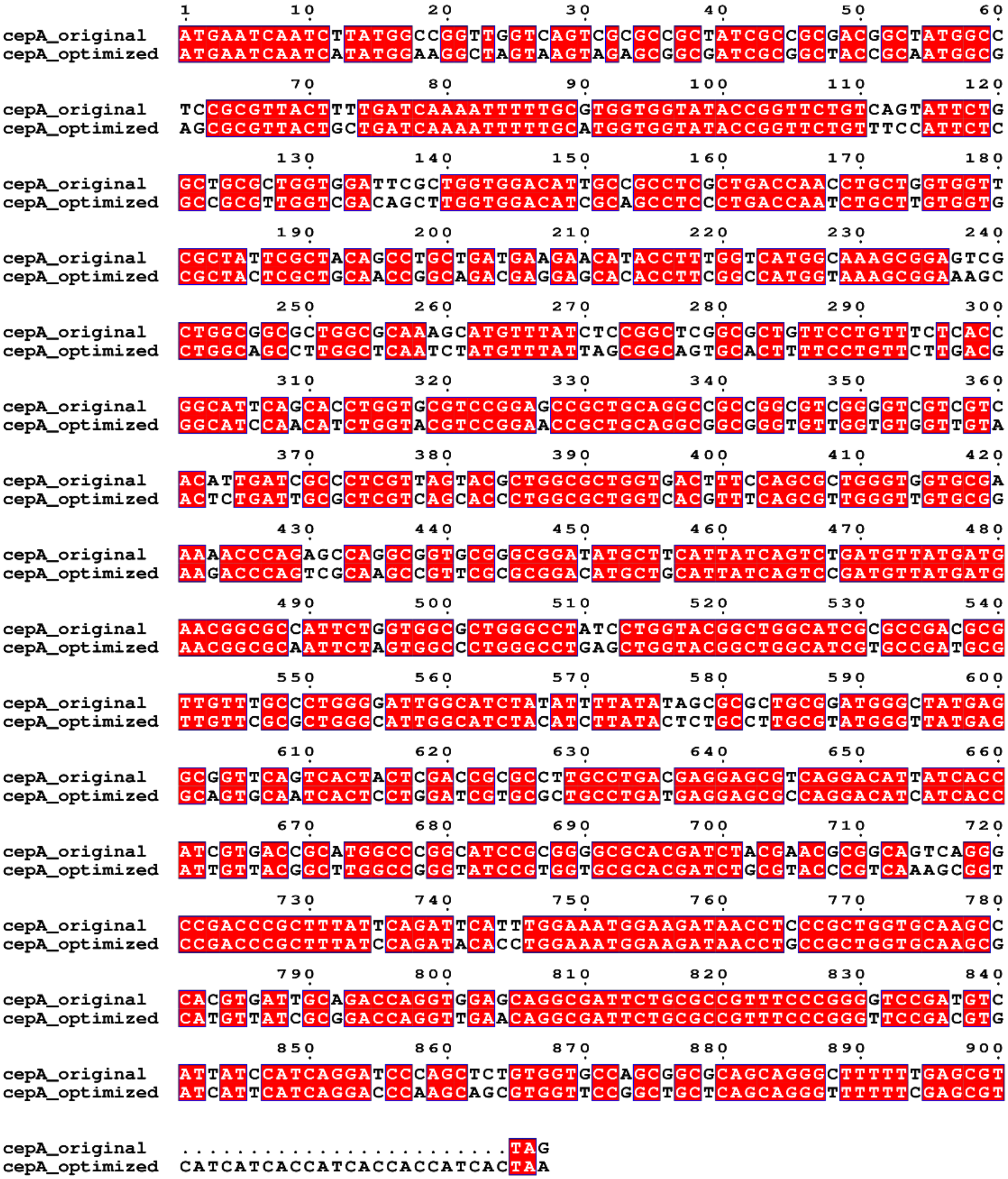

Figure S4. Alignment of the original sequence of *cepA* (cepA_original) taken from *Klebsiella pneumoniae* and the codon-optimized sequence of *cepA* (cepA_optimized) modified for the expression in *E. coli* with an 8xHis-tag sequence included.

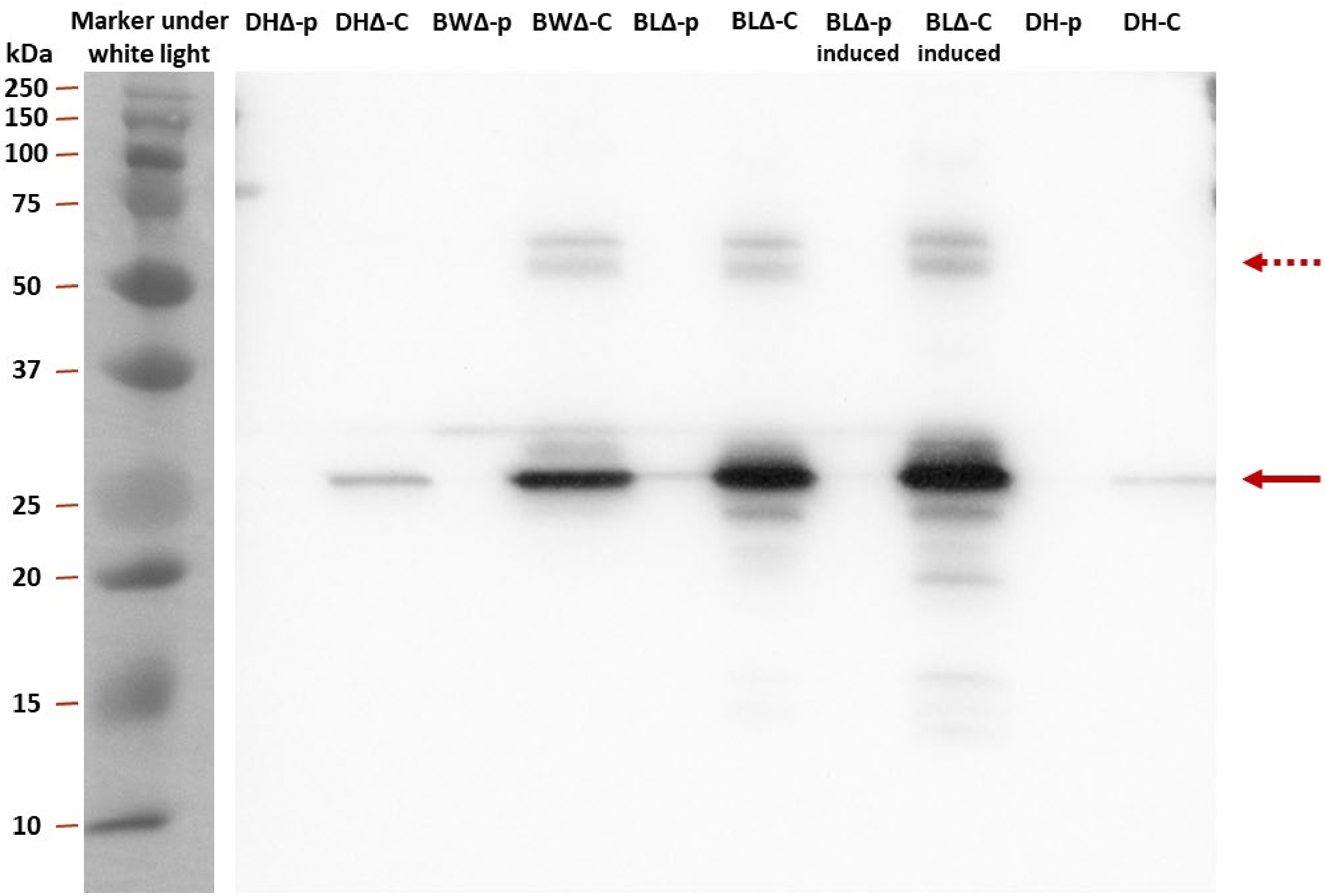

Figure S5. Detection of CepA by a Western Blot. Total membrane fractions (45 µg) isolated from different bacterial strains were probed using a His-tag specific antiserum. The position of the predicted size of the His-tagged CepA (32.9 kDa) is marked by a solid arrow. The faint band of CepA homodimer (65.8 kDa) is marked by a dotted arrow.

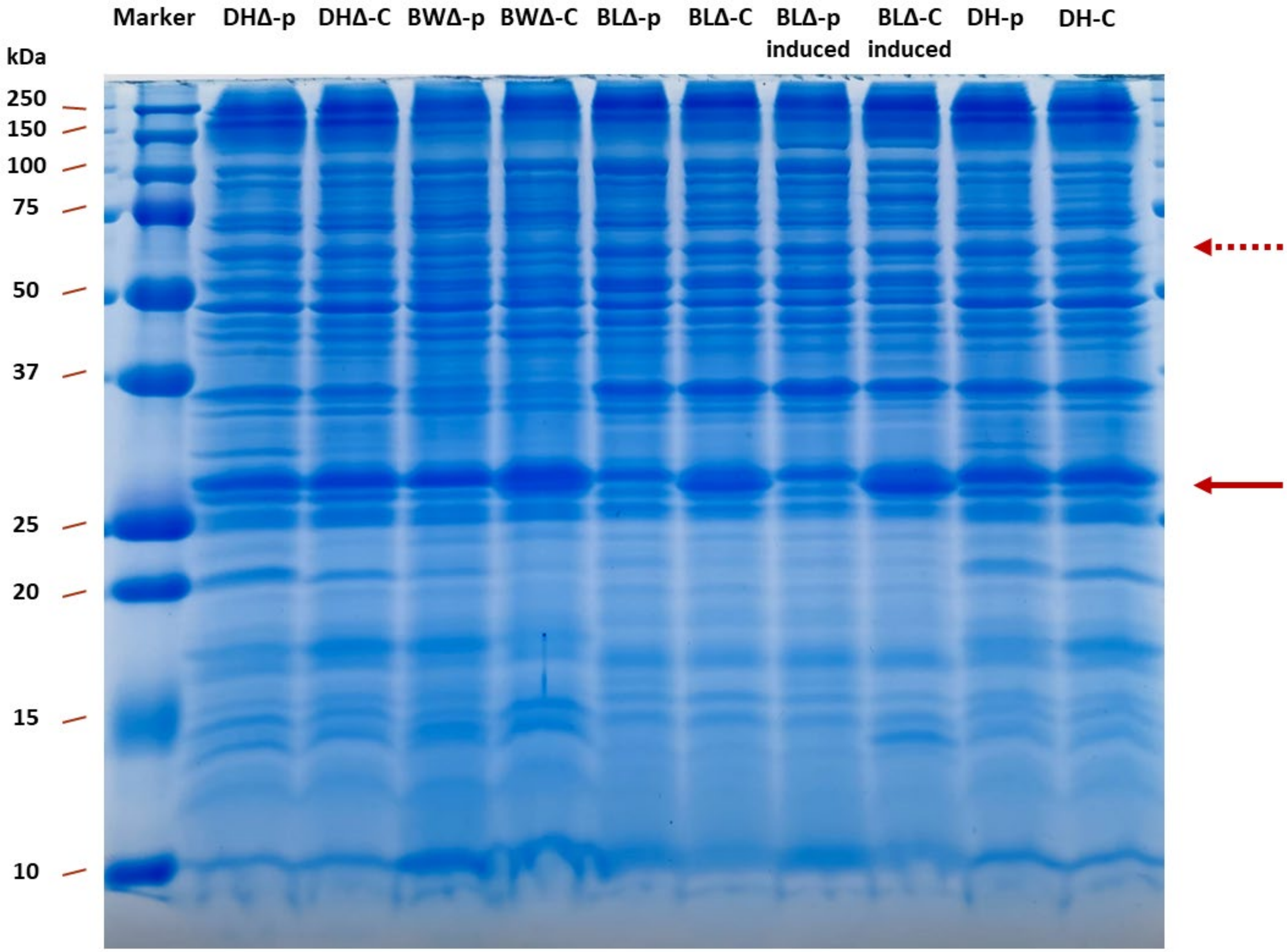

Figure S6. Commasine-stained SDS-PAGE gel of 45 µg of total membrane fraction samples. The position of the predicted size of the His-tagged CepA (32.9 kDa) is marked by a solid arrow. The likely band of CepA homodimer (65.8 kDa) is marked by a dotted arrow.

Table S2. List of *E. coli* strains used in this study and their notable features

Strain	Feature	Origin
E. coli DH5α	F⁻ *endA1 glnV44 thi-1 recA1 relA1 gyrA96 deoR nupG purB20* φ80d*lacZ*ΔM15 Δ(*lacZYA-argF*)U169, hsdR17($r_K^- m_K^+$), λ⁻	(1)
E. coli BL21(DE3)	F⁻ *ompT gal dcm lon hsdS$_B$*($r_B^- m_B^-$) λ(DE3 [*lacI lacUV5-T7p07 ind1 sam7 nin5*]) [*malB*⁺]$_{K\text{-}12}$(λ^S)	(2)
E. coli BW25113	Δ*(araB–D)567* Δ*(rhaD–B)568* Δ*lacZ4787*(::*rrnB-3*) *hsdR514 rph-1*	(3)
E. coli DH5α Δ*acrAB*	*E. coli* DH5α with the AcrAB efflux pump system inactivated, hypersensitive to antimicrobials.	Previously generated in our lab
E. coli BL21(DE3) Δ*acrAB*	*E. coli* BL21(DE3) with the AcrAB efflux pump system inactivated, hypersensitive to antimicrobials.	Previously generated in our lab
E. coli BW25113 Δ*acrAB*	*E. coli* BW25113 with the AcrAB efflux pump system inactivated, hypersensitive to antimicrobials.	(4)
E. coli DH5α (pBS)	*E. coli* DH5α carrying pBlueScript II SK (+) vector, ampicillin resistance	This study
E. coli DH5α Δ*acrAB* (pBS)	*E. coli* DH5α Δ*acrAB* carrying pBlueScript II SK (+) vector, ampicillin resistance	This study
E. coli DH5α Δ*acrAB* (pBS-CepA)	*E. coli* DH5α Δ*acrAB* carrying pBlueScript II SK (+) vector with a codon-optimized *cepA* gene inserted, ampicillin resistance	This study
E. coli BL21(DE3) (pBS)	*E. coli* BL21(DE3) carrying pBlueScript II SK (+) vector, ampicillin resistance	This study
E. coli BL21(DE3) Δ*acrAB* (pBS)	*E. coli* BL21(DE3) Δ*acrAB* carrying pBlueScript II SK (+) vector, ampicillin resistance	This study
E. coli BL21(DE3) Δ*acrAB* (pBS-CepA)	*E. coli* BL21(DE3) Δ*acrAB* carrying pBlueScript II SK (+) vector with a codon-optimized *cepA* gene inserted, ampicillin resistance	This study
E. coli BW25113 (pBS)	*E. coli* BW25113 carrying pBlueScript II SK (+) vector, ampicillin resistance	This study
E. coli BW25113 Δ*acrAB* (pBS)	*E. coli* BW25113 Δ*acrAB* carrying pBlueScript II SK (+) vector, ampicillin resistance	This study
E. coli BW25113 Δ*acrAB* (pBS-CepA)	*E. coli* BW25113 Δ*acrAB* carrying pBlueScript II SK (+) vector with a codon-optimized *cepA* gene inserted, ampicillin resistance	This study

REFERENCES

1. Hanahan D. 1983. Studies on transformation of *Escherichia coli* with plasmids. J Mol Biol 166:557-580.
2. Studier FW, Moffatt BA. 1986. Use of bacteriophage T7 RNA polymerase to direct selective high-level expression of cloned genes. J Mol Biol 189:113-130.
3. Datsenko KA, Wanner BL. 2000. One-step inactivation of chromosomal genes in *Escherichia coli* K-12 using PCR products. Proc Natl Acad Sci U S A 97:6640-6645.
4. Ohene-Agyei T, Lea JD, Venter H. 2012. Mutations in MexB that affect the efflux of antibiotics with cytoplasmic targets. FEMS Microbiol Lett 333:20-27.
